# Recruitment of orbitofrontal cortex during unpredictable threat among adults at risk for affective disorders

**DOI:** 10.1002/brb3.757

**Published:** 2017-07-11

**Authors:** Namik Kirlic, Robin L. Aupperle, Masaya Misaki, Rayus Kuplicki, Ruben P. Alvarez

**Affiliations:** ^1^ Laureate Institute for Brain Research Tulsa OK USA; ^2^ Department of Community Medicine University of Tulsa Tulsa OK USA

**Keywords:** anxiety, behavioral inhibition, depression, high risk, orbitofrontal cortex, threat

## Abstract

**Background:**

Mood and anxiety disorders are characterized by altered prefrontal‐amygdala function and increased behavioral inhibition (BI) in response to potential threat. Whether these alterations constitute a vulnerability or a symptom of illness remains unclear. The medial orbitofrontal cortex (mOFC) is thought to play a central role in estimating probability and cost of threat, in turn informing selection of subsequent behaviors. To better understand the behavioral and neural processes that may be associated with risk for psychopathology, we used a virtual reality paradigm to examine behavioral and neural responses of psychiatrically healthy adults with familial history of affective disorders during anticipation of unpredictable threat.

**Methods:**

Twenty psychiatrically healthy adults with high familial risk for affective disorders and 20 low‐risk matched controls underwent functional magnetic resonance imaging concurrent with a paradigm in which they explored virtual contexts associated with the threat of shock or safety from shock. Subjective anxiety ratings, skin conductance, exploratory behavior, and neural responses were examined for threat versus safe conditions.

**Results:**

High‐risk adults evidenced greater right mOFC activation, as well as greater BI, compared to low‐risk adults. There were no significant group differences in subjective ratings or autonomic responses. Individuals exhibiting greater activity in the right mOFC showed greater BI and decreased skin conductance response.

**Conclusions:**

These results suggest that BI and mOFC recruitment during anticipation of aversive outcomes may reflect a vulnerability for affective disorders. However, such a response may also serve as a compensatory response, protecting these high‐risk individuals from negative outcomes (i.e., increased physiological arousal). These results suggest that the OFC may play a central role in driving threat‐related behaviors and thus may be a target for efforts aimed at early detection or prevention.

## 1 INTRODUCTION

Behavioral inhibition (BI) is characterized by a heritable and stable fearful and cautious disposition, as well as a propensity to respond anxiously in novel circumstances and with hesitation, reduction in activity, and avoidance during anticipation of potentially threatening situations (Clauss, Avery, & Blackford, 2015; Kagan, Reznick, & Snidman, 1987; Robinson, Kagan, Reznick, & Corley, 1992). When exaggerated and contextually inappropriate, BI is among the strongest predictors of psychopathology, including not only anxiety but also depression (Caspi, 1996; Clauss & Blackford, 2012; Clauss et al., 2015; Gladstone & Parker, 2006; Hirshfeld‐Becker et al., 2008). Furthermore, there is some evidence that BI is more common among individuals with family history of mental illness and compounds the risk for psychopathology (Hirshfeld‐Becker et al., 2008). Nevertheless, not all behaviorally inhibited individuals report elevated states of anxiety or go on to develop anxiety and mood disorders, which suggests that, for some, avoidance of threatening situations may serve as an adaptive, resilient mechanism through which negative affective states are regulated (Fowles, 1987).

An important aspect of BI is how individuals respond to the anticipation of potentially aversive events. Understanding the processes involved in the anticipation of aversive events has been less of a focus for research on depression than for anxiety. However, given that BI may serve as a risk factor for depression and that depression and anxiety are highly comorbid (Barlow, 2002; Kessler, Merikangas, & Wang, 2007; Lamers et al., 2011), it is likely that aberrant responses during anticipation of aversive events may serve as a mechanism for the development and maintenance of mood and anxiety disorders in general (Sandi & Richter‐Levin, 2009).

Neuroimaging studies have provided abundant knowledge concerning the networks involved in the anticipation of potentially aversive events. This research highlights neurocircuits involving the orbitofrontal cortex (OFC), insula, ventromedial prefrontal cortex (vmPFC), amygdala, bed nucleus of the stria terminalis (BNST), and anterior midcingulate cortex (aMCC) (Alvarez et al., 2015; Grupe & Nitschke, 2013; Nitschke, Sarinopoulos, Mackiewicz, Schaefer, & Davidson, 2006). The medial prefrontal cortex (mPFC) has been strongly linked to emotional processing and control of emotional behaviors (Etkin, Egner, & Kalisch, 2011; Phillips, Ladouceur, & Drevets, 2008), while animal research suggests that the right mPFC has a direct role in modulation of stress‐regulatory circuits (Sullivan & Gratton, 2002). The OFC in particular may be critical for guiding appropriate responses under such circumstances by signaling predictions about specific outcomes associated with sensory events, choices, or actions while taking into account the current state of the organism (Rudebeck & Murray, 2014). It is believed that it does so by encoding and storing the relative, expected values of future events, and importantly, updating this valuation based on experienced outcomes. Specifically, while the role of the lateral OFC (lOFC) is to assess what potential alternative choices are available, the medial OFC (mOFC) is thought to execute the value‐based comparison between them in order to direct choice behavior (Rudebeck & Murray, 2014). This information is then relayed to subcortical structures that subsequently direct autonomic and behavioral responses (Rudebeck & Murray, 2011, 2014). Therefore, during anticipation of threat, the mOFC may estimate the probability and cost of threat, and in turn, inform the selection of the appropriate autonomic and behavioral responses based on these estimates.

Disrupted functioning of the OFC has been implicated in mood and anxious pathology, as well as in BI. For example, depressed patients exhibit increased activity in the OFC during exposure to negatively valenced stimuli, as well as during rest (Drevets, 2007), and lesions of the OFC increase the risk for developing depression (MacFall, Payne, Provenzale, & Krishnan, 2001). Similarly, evidence of failure to recruit the mOFC to appropriately inhibit anxiety responses has been observed across a range of anxiety disorders (Milad & Rauch, 2007). Finally, OFC has also been identified as a neural substrate of inhibited (i.e., anxious) temperament, with observed volumetric and functional differences that may reflect disruption in inhibitory connections to amygdala (Clauss et al., 2015). This includes findings of hemispheric asymmetry in both structure and function of this region, such that reactive temperament in early childhood predicts right frontal activation as measured by EEG in adolescence (McManis, Kagan, Snidman, & Woodward, 2002) and greater right mOFC volume in adulthood (Schwartz et al., 2010), as well as that high risk for alcohol dependence predicts greater right mOFC among adolescents (Hill, Tessner, Wang, Carter, & McDermott, 2010).

It is still unclear how alterations in neural networks implicated in research on anxiety, depression, and BI lead to the development of psychopathology. Research involving healthy individuals at risk of developing mood and anxiety disorders by virtue of family history removes the confound of current symptomatology. Such studies are therefore useful for identifying not only aberrations or dysfunctions that may serve as risk factors, but may also point to protective mechanisms. Most of these studies have focused on at‐risk children and adolescents and report alterations in medial prefrontal‐amygdala‐striatal networks that are similar to those implicated in affective disorders. Enhanced anticipatory anxiety as well as overall greater startle responses have been found in adolescent offspring of parents with anxiety disorders relative to low‐risk controls (Grillon, Dierker, & Merikangas, 1998). Greater autonomic responses during conditioning, as well as while anticipating threat during extinction, have also been observed in at‐risk children (Craske et al., 2008). Furthermore, neuroimaging studies with at‐risk children have shown increased responses in the lateral OFC and insula to aversive stimuli (McCabe, Woffindale, Harmer, & Cowen, 2012), dorsal anterior cingulate cortex (ACC) to losses (Gotlib et al., 2010), pregenual ACC to negative words (Mannie et al., 2008), and ventrolateral prefrontal cortex (vlPFC) during negative mood induction (Joormann, Cooney, Henry, & Gotlib, 2012), as well as diminished responses in dorsolateral prefrontal cortex (dlPFC) to fearful faces (Mannie, Taylor, Harmer, Cowen, & Norbury, 2011). Increased amygdala responses during negative moods (Joormann et al., 2012) and to fearful faces (Chai et al., 2015; Monk et al., 2008) have been observed in HR adolescents relative to controls, although no differences to fearful faces have also been reported (Mannie et al., 2011). Temperamentally inhibited children fail to engage the mPFC and ACC during anticipation of threat (Clauss, Benningfield, Rao, & Blackford, 2016), while young adults who self‐identify as temperamentally inhibited show greater hemodynamic responses in dlPFC and dorsal and rostral ACC during anticipation of threat and no differences in the amygdala as compared to uninhibited young adults (Clauss et al., 2014).

Although these studies suggest distinct behavioral and neural alterations in at‐risk individuals, the overwhelming focus on children and adolescents does not tell us whether and how might these alterations translate into adulthood promoting risk or resilience. Furthermore, although the onset of anxiety disorders is typically much earlier, the median onset for mood disorders is 32 years of age, with interquartile range between 19 and 44 (Kessler et al., 2007). Thus, healthy young adults with a family risk of affective disorders may continue to be at risk for psychopathology.

This study examined OFC and behavioral responses to anticipation of unpredictable threat (AUT) in psychiatrically healthy adults with familial history of mood disorders (HR) and an age‐ and gender‐matched cohort with no family history of psychopathology (LR). We used the AUT task (Alvarez et al., 2015), in which unpredictable shock is presented in one virtual reality environment, while in the other, no shock is delivered. Relative to passive viewing or anticipation of emotional stimuli, this paradigm's use of physically salient stimuli during behavioral exploration of virtual environments arguably represents a more relevant threat to subjects and objective assessment of inhibited behavior, thereby more closely reflecting real‐life situations. We hypothesized that the HR group would exhibit greater BI than the LR group, as measured by amount of time spent exploring the threat relative to safe condition. We further hypothesized that, relative to LR, the HR group would exhibit heightened activation in the mOFC during anticipation of threat relative to safe condition. We predicted activity in the mOFC would positively relate to BI and skin conductance responses (SCR). Finally, given the role of BNST in AUT (Alvarez et al., 2015; Davis, Walker, Miles, & Grillon, 2010; Grupe & Nitschke, 2013), we also explored differences between groups in BNST.

## 2 MATERIALS AND METHODS

### 2.1 Participants

Forty (24 females; mean age = 29.65 years, *SD *= 7.95) medically healthy volunteers, ages 18 through 50 years, participated in this study, including 20 psychiatrically healthy individuals with a first‐degree relative with a mood disorder (HR), and 20 age‐ and gender‐matched individuals with no family history of mood disorders (LR). Participants’ psychiatric status was determined with a Structured Clinical Interview for DSM‐IV‐TR Axis I Disorders (SCID‐I; First, Spitzer, Gibbon, & Williams, 2002). Family psychiatric history was established via the Family Interview for Genetic Studies (Maxwell, 1992). Exclusion criteria were past or current psychiatric disorders as per SCID‐I, major neurological (including history of TBI) or medical disorders, substance abuse, current pregnancy, and history of psychotropic medication use or other drugs likely to influence cerebral function or blood flow within 3 weeks. Participants with a body mass index greater than 35 kg/m^2^ were also excluded from the study in order to minimize difficulty obtaining electromyographic recordings during individual pain threshold testing. Participants provided informed consent and received monetary compensation for their participation. All study procedures were approved by the Western Institutional Review Board. For further details, see Appendix S1.

### 2.2 Experimental procedures and materials

#### 2.2.1 General procedure

Prior to scanning, participants received task instructions, completed questionnaires, and underwent sensor application. Participants next completed nociceptive flexion reflex/pain threshold testing (see Appendix S1) and practiced navigating a nontask virtual context for 2 min in a full‐scale mock MRI scanner under no threat of receiving stimulation. Next, participants underwent anatomical and resting‐state scans, followed by a 4‐min practice scan during which they explored task contexts according to task instructions (receiving a single electric stimulation in the threat context). Participants then completed a contingency awareness test to assess the accurate understanding of experimental contingencies, which was followed by task scans.

#### 2.2.2 Psychological assessment

A clinician‐administered Hamilton Rating Scale for Depression was used to assess depressive symptomatology, while the self‐report State‐Trait Anxiety Inventory was used to assess both trait anxiety proneness (STAI‐T) and state feelings of anxiety or apprehension in response to current circumstances (STAI‐S) (Spielberger, 1983).

#### 2.2.3 Anticipation of unpredictable threat task

The AUT task was conducted as described in previous publications (Alvarez et al., 2015) (see Appendix S1). Briefly, participants were informed that they were to virtually explore two computer‐simulated rooms and that later they would be asked what they learned about each room. Participants were instructed that when in the purple (or peach, counterbalanced across participants) room, they could receive a stimulation on the ankle at any time (threat context), but when they were in the peach (or purple) room they would never receive a stimulation (safe context). Therefore, it was expected that throughout the task participants would engage in sustained AUT during threat relative to safe contexts.

**Figure 1:**
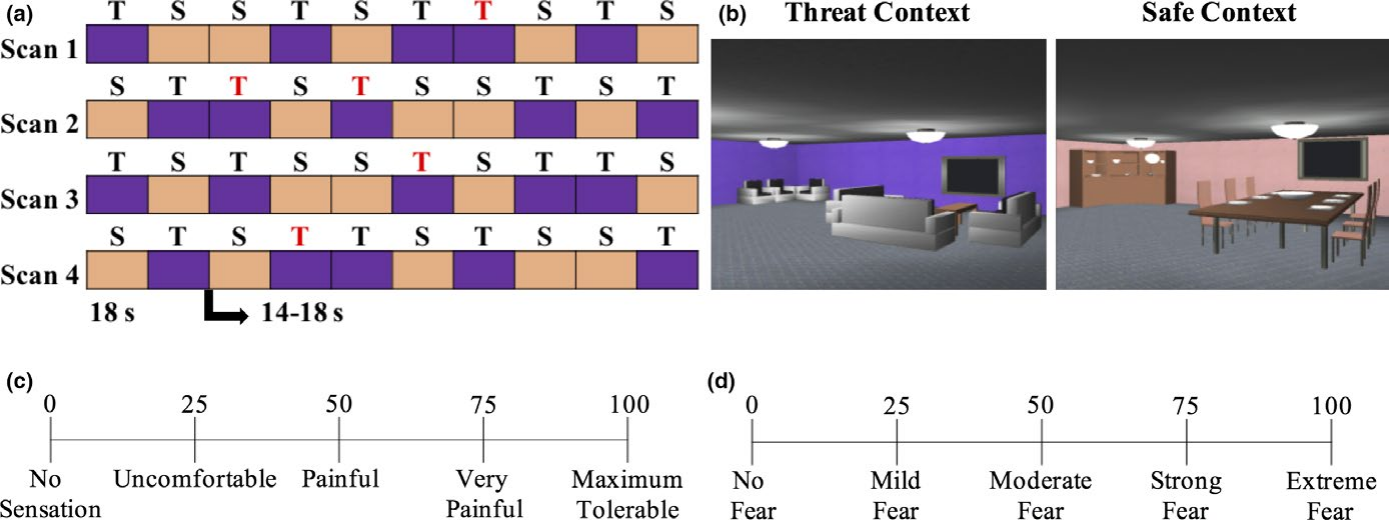
Anticipation of unpredictable threat task (AUT). (a) During the task, participants explored two contexts, one in which there was a threat of receiving a transcutaneous stimulation at any time (T), and one in which they were safe from receiving any stimulation (S). The acronyms colored in red denote contextual epochs in which unsignaled electrical stimulations were administered. (b) Static pictures of the computer‐simulated rooms that served as threat and safe contexts. (c) Following each threat context in which an electric stimulus was administered, participants rated the intensity of the stimulus received. (d) Following each scan, participants also retrospectively rated how fearful they were in the threat and safe contexts using a 0–100 scale

During each of four fMRI scans (each 350 s), five threat and five safe contexts were semirandomly presented for a duration of 18 s followed by an interstimulus interval (ISI) of 14–18 s. Order of scan presentation was counterbalanced across participants. One to two unpredictable (i.e., unsignaled) electrical stimulations (unconditioned stimulus [US]) were delivered during threat contexts (range 3–16 s postcontext onset; mean onset=9.6 s) during each fMRI scan for a total of 5. No US was administered during safe contexts. Following each threat context that included electric stimulation, participants rated the intensity of the stimulus received during the ISI. Postscan, participants retrospectively rated how fearful they were in each context (Figure 1).

Behavioral inhibition under conditions of sustained AUT was indexed as the average time spent exploring the threat relative to the safe context (smaller numbers indicated less exploratory behavior, or greater BI). BI has been widely used in animal models of anxiety, and can be assessed as the decreased duration of time the animal spends in a novel environment, such as in open field tests (Kumar, Bhat, & Kumar, 2013). We calculated the exploratory behavior metric (i.e., total time spent pressing forward/backward buttons) as the total time spent pressing any button minus total time spent pressing left and right buttons. Exploratory behavior is distinct from locomotor behavior, and animal and human research on exploration suggests that decreased exploratory activity, rather than overall motor activity (e.g., orientation toward stimulus), may be associated with anxiety‐related responses (Geyer, Russo, & Masten, 1986; Young, Minassian, Paulus, Geyer, & Perry, 2007; Walz, Mühlberger, & Pauli, 2016). Therefore, because pressing left/right buttons enabled participants to orient left/right but not advance forward/backward, it was hypothesized that the latter metric would best index exploratory behavior.

#### 2.2.4 Electric stimuli, skin conductance, and physiological monitoring

Electric stimulations were delivered with a Digitimer DS7A (Hertfordshire, UK) constant current stimulator triggered by a presentation computer and waveform generator (Agilent 33220A; Santa Clara, CA, USA) using two MRI‐compatible Ag‐AgCl‐stimulating surface electrodes (2 cm interelectrode distance) attached to the left ankle over the retromalleolar pathway of the sural nerve, 2 cm posterior to the malleolus (Roy, Piche, Chen, Peretz, & Rainville, 2009). Task stimulus intensity was set at 1.2 times the level obtained during threshold testing, but never exceeding 40 mA for the safety of the participant. SCR were recorded using MRI‐compatible Ag‐AgCl electrodes placed on the medial side of the right foot over the abductor hallucis muscle (Fowles et al., 1981), and using a Biopac Systems electrodermal activity module. Offline data analysis of SCRs was performed using the general linear convolution model‐based analysis of waveforms as implemented in SCRalyze software (2.1.6b, scralyze.sourceforge.net) to estimate the mean response amplitude for threat and safe conditions (Bach, Daunizeau, Friston, & Dolan, 2010; Bach, Flandin, Friston, & Dolan, 2009). For further details, see Appendix S1.

### 2.3 Imaging procedures and data analysis

#### 2.3.1 Data acquisition and imaging parameters

Functional and structural images were acquired using a Discovery MR750 whole‐body 3.0 Tesla MRI scanner (GE Healthcare, Milwaukee, WI, USA). A receive‐only 32‐element phased array coil (Nova Medical Inc., Wilmington, MA, USA) optimized for parallel imaging was used for MRI signal reception. We acquired high‐resolution functional scans in order to successfully image small structures like the BNST, whose size and location can be challenging to study in humans using fMRI (Alvarez, Chen, Bodurka, Kaplan, & Grillon, 2011; Hennigan, D'Ardenne, & McClure, 2015).

Functional blood oxygenation‐level‐dependent (BOLD) scans used a single‐shot, gradient‐recalled echo‐planar imaging (EPI) sequence with sensitivity encoding (96 × 96 matrix, 240 mm field of view (FOV), 2.5 × 2.5 mm^2^ in‐plane resolution, 35 axial slices, 2.9 mm slice thickness with 0.5 gap, 200 ms repetition time (TR), 25 ms echo time (TE), 40° flip angle, 250 kHz sampling bandwidth, 175 volumes, and SENSE acceleration factor *R* = 2 in the phase‐encoding direction). EPI images were reconstructed into a 128 × 128 matrix, with 1.875 × 1.875 × 2.9 mm voxel size. One T1‐weighted Magnetization Prepared Rapid Gradient Echo (MPRAGE) imaging sequence with SENSE was used for anatomical reference and alignment purposes (256 × 256 matrix size, 240 mm FOV, 0.938 × 0.938 mm^2^ in‐plane resolution, 1.1 mm slice thickness, 5 ms TR, 1.95 ms TE, 8° flip angle, 31.25 kHz sampling bandwidth, 134 axial slices per volume, and acceleration factor *R* = 2).

#### 2.3.2 Data preprocessing and subject‐level analyses

Functional image preprocessing and analysis were performed using AFNI (AFNI: RRID:SCR_005927) (http://afni.nimh.nih.gov/afni). Advanced Normalization Tools (ANTS: RRID:SCR_004757) was used to optimize spatial alignment of functional data to the TT_N27 T1‐weighted template (see Appendix S1). The first five volumes were discarded and slice timing correction was performed for each volume. The anatomical image was aligned to the first EPI image (using align_epi_anat.py), resampled to the same resolution as the EPI image, and warped to the TT_N27 T1‐weighted template using ANTs. EPI images were realigned to the first volume and then normalized to the template image using the anatomy to template warping parameters. EPI data were smoothed with a small 1.875 mm FWHM Gaussian kernel. Signal intensity was normalized to reflect percent signal change (PSC) from the mean intensity of each voxel across the time course.

Single subject‐level analyses were conducted using AFNI's 3dREMLfit. The regression model included regressors for each task context, as well as regressors of noninterest to account for head motion (roll, pitch, yaw, superior, left, and posterior; demeaned and derivative values), end‐tidal CO2, cubic trend to eliminate slow signal drifts, and time points including electric stimulations, stimulation intensity ratings, fixation color changes, and navigation behavior. To give the shape of the BOLD response maximum flexibility, task contexts were modeled as the sum of piecewise linear B‐spline basis functions or tent functions. Fifteen tent functions covering 30s were used to account for the full context duration (0–18 s) and subsequent BOLD response recovery. For the contrast of threat versus safe context, voxel‐wise analysis included only regressors for the 10 time points (0–18 s) spanning each context. The first time point was assumed to have zero magnitude to account for the expected delay in the BOLD response to context onset. Finally, the analysis was designed in such a way that the results were not biased by the delivery of US. Specifically, responses to threat context included only the trials in which shock was not delivered. Only safe context trials closest in proximity to the unreinforced threat trials were used in order to allow for an equal number of trials in the threat versus safe contrast. Therefore, the final analysis included 15 threat and 15 safe context trials.

#### 2.3.3 Group analyses

Behavioral and demographic analyses were carried out using SPSS 22. We used independent‐samples *t* tests to assess for differences in demographic variables and BI between HR and LR groups. To examine SCRs and subjective anxiety ratings between participant groups across conditions we used a repeated‐measures ANOVA. All tests were two‐tailed and considered significant at *p *<* *.05.

Whole‐brain voxel‐wise analysis for the threat versus safe context adjusting for subject age was conducted using AFNI's 3dMEMA program (Chen, Saad, Nath, Beauchamp, & Cox, 2012). The results were corrected for multiple comparisons using Monte Carlo simulations. Given recent concerns that typical family‐wise error (FWE) threshold of *p *<* *.05 may not adequately control for false positive inferences (Eklund, Nichols, & Knutson, 2015), significance criterion for detecting activation for the whole‐brain analysis in the full sample was set at a corrected FWE rate of *p *<* *.005 (cluster size ≥ 16 voxels), thresholded per‐voxel at *p *<* *.0001 for a meaningful separation of clusters, determined using AFNI program 3dClustSim. Whole‐brain analysis was also conducted contrasting groups (HR, LR) on the mean PSC for threat versus safe contexts, with significance criterion set at a corrected FWE rate of *p *<* *.005, thresholded per‐voxel at *p *<* *.001 (cluster size ≥ 30 voxels).

Group differences in bilateral mOFC regions for the threat versus safe contrast were performed on the data extracted from their respective anatomical regions of interest (ROIs) separately, defined a priori using prerendered stereotaxic masks available in AFNI using the Talairach and Tournoux (1988) N27 atlas brain (Left mOFC: *x* = −12, *y* = 20, *z* = −9 [59 voxels]; Right mOFC: *x* = 12, *y* = 20, *z* = −9 [63 voxels]; Left BNST: *x* = −8, *y* = 2, *z* = 6 [19 voxels]; Right BNST: *x* = 8, *y* = 2, *z* = 6 [19 voxels]; Figure S1). To account for multiple comparisons, we used a Bonferonni correction of *p *<* *.013. The temporal signal‐to‐noise ratio (TSNR) of 77.86 (*SD* = 24.26) and 95.86 (*SD *= 18.05) for mOFC and BNST, respectively, was well above the recommended 40 (Murphy, Bodurka, & Bandettini, 2007), suggesting a reliable detection of signal within these regions.

For analyses examining the relationship between brain regions and behavioral measures, the average beta values for threat versus safe context were extracted per individual from each predetermined anatomical ROI. All analyses included only threat trials in which no US was delivered and a comparable number of safe trials.

## 3 RESULTS

### 3.1 Demographic, behavioral, and skin conductance response data

Demographic data are shown in Table 1. There were no significant differences between LR and HR groups in age, body mass index, or mood and anxiety symptoms (*p *>* *.05). As compared to LR, the HR group exhibited greater BI, that is, less exploratory behavior during threat versus safe contexts [HR: *M(SD) *= −0.71(1.21); LR: *M(SD) *= 0.12(1.06); *t*_(38)_=2.31, *p *<* *.05]. On average, LR and HR participants rated the US intensity as moderately painful [HR: *M(SD) *= 49.40(14.51); LR: *M(SD) *= 47.28(9.01); *t*_(38)_ = 0.56, *p *=* *.58]. For subjective anxiety ratings, there was a main effect of Condition [*F*(1,38) = 66.30, *p *<* *.001, η^2^ = 0.64], but not Group [*F*(1,38) = 1.92, *p *=* *.17, η^2^ = 0.05], or a Group x Condition interaction [*F*(1,38) = 2.39, *p *=* *.13, η^2^ = 0.06]. Subjective anxiety was greater during threat than safe contexts for all participants [Threat: *M(SD) *= 30.25(23.36); Safe: *M(SD) *= 1.63(3.39); *t*_(39)_ = 8.01, *p *<* *.001]. Similarly, analyses with SCRs revealed a main effect of Condition [*F*(1,38) = 24.95, *p *<* *.001, η^2^ = 0.40], but not Group [*F*(1,38) = 1.08, *p *=* *.31, η^2^ = 0.03], or a Group x Condition interaction [*F*(1,38) = 2.46, *p *=* *.13, η^2^ = 0.06]. SCRs were greater during threat than safe context [Threat: *M(SD) *= 0.03(0.03); Safe: *M(SD) *= 0.01(0.02); *t*_(39)_  = 7.10, *p *<* *.001]. BI was not associated with subjective anxiety ratings [*r*_(38)_ = −.11, *p = *.50], but related to SCRs [*r*_(38)_ = .40, *p < *.05].

**Table 1:** Descriptive and inferential statistics for demographic and questionnaire data for each group

Variable	Low risk (*n *=* *20)		High risk (*n *=* *20)		*t*_(38)_	*p*‐value
	*M*	*SD*	*M*	*SD*		
Demographics						
Age (years)	29.80	7.85	29.50	8.26	0.12	0.907
BMI (kg/m^2^)	26.16	4.79	24.40	4.56	1.19	0.243
Mood and anxiety symptoms						
HAM‐D	0.70	1.22	1.65	2.43	1.561	0.127
STAI‐S	24.65	4.99	25.90	5.42	0.759	0.452
STAI‐T	25.35	4.07	26.80	5.76	0.920	0.363

BMI, body mass index; HAMD, Hamilton Rating Scale for Depression; STAI, state‐trait anxiety inventory.

### 3.2 Imaging results

**Figure 2:**
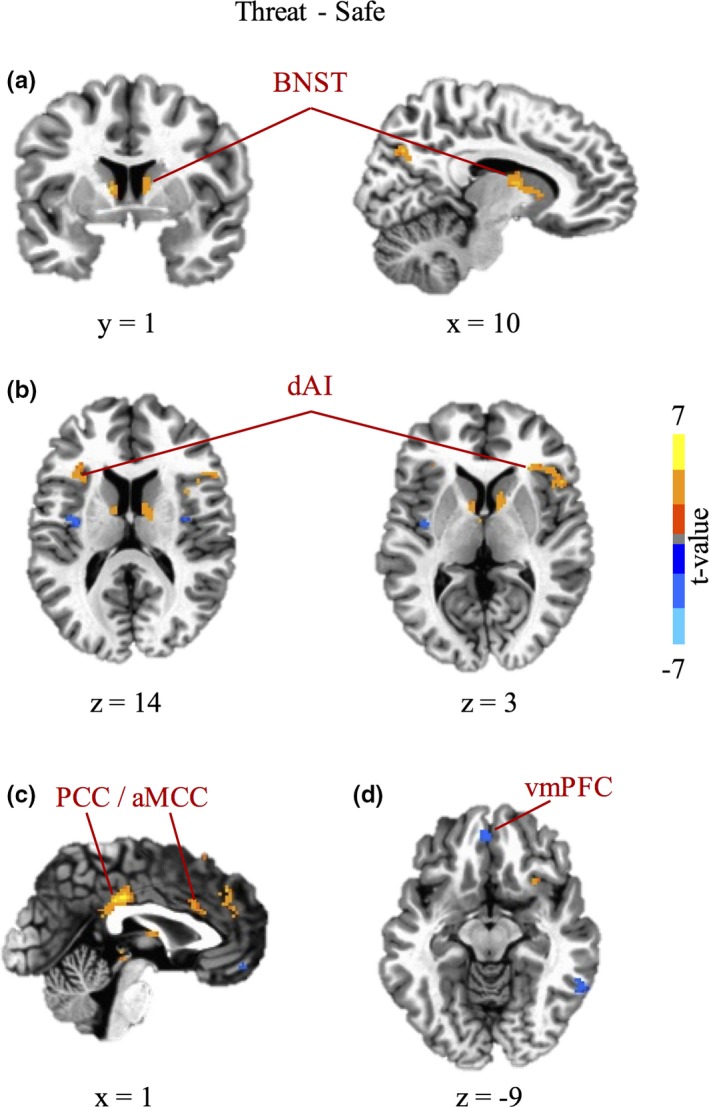
During anticipation of unpredictable threat, subjects exhibited increased hemodynamic activity in (a) left and right bed nucleus of stria terminalis (BNST), (b) left and right dorsal regions of anterior insula (dAI), (c) right anterior midcingulate cortex (aMCC) and posterior cingulate cortex (PCC), and decreased hemodynamic activity in (d) ventromedial prefrontal cortex (vmPFC). Results shown were corrected for multiple comparisons at *p*_corr_ < .005. Left is left

Whole‐brain voxel‐wise analyses for the study sample in general revealed greater activation during the threat context as compared to the safe context in left and right BNST, dorsal and ventral regions of the anterior insula, and aMCC, among other regions (Table 2; Figure 2). In addition, several regions exhibited greater activation during the safe context than the threat context, including the vmPFC.

**Table 2:** Regions of the brain showing differences in the hemodynamic response for Threat > Safe and Safe > Threat for all participants

Hemisphere/location	Peak coordinates^a^			*t*_(38)_	No. of voxels
	*x*	*y*	*z*		
Threat > Safe					
L posterior cingulate gyrus	−1	−27	32	10.18	197
R dorsal anterior insula/inferior frontal gyrus	44	22	3	7.18	172
L bed nucleus of stria terminalis	−8	1	8	6.81	103
R bed nucleus of stria terminalis	10	−3	12	6.99	85
L dorsal anterior insula	−33	20	14	6.41	55
L precuneus	−8	−68	31	7.45	49
R medial superior frontal gyrus	3	35	31	7.51	47
R precuneus	10	−68	31	7.44	47
R ventral anterior insula	29	18	−7	7.98	33
R lateral superior frontal gyrus	25	52	19	6.82	28
R anterior midcingulate gyrus	1	14	29	5.73	26
R superior frontal gyrus, medial	3	16	57	6.27	21
R medial superior frontal gyrus	3	38	21	5.57	21
R paraventricular thalamic nucleus	3	−27	1	5.46	20
R anteromedial insula	37	8	12	5.20	19
Safe>Threat					
R postcentral gyrus	53	−20	42	6.58	63
L precentral gyrus	−59	−12	27	5.86	44
L posteromedial insula	−35	−8	8	6.16	42
L paracentral lobule	−3	−37	63	6.45	37
L ventromedial prefrontal cortex	−3	44	−9	6.20	36
R middle temporal gyrus	59	−52	−7	5.87	33
L precentral gyrus	−46	−10	36	5.37	23
R posteromedial insula	38	−7	12	6.15	17

L, left; R, right. The *x*,* y*,* z* coordinates indicate distance in millimeters from the anterior commissure in three dimensions: *x*, right to left; *y*, anterior to posterior; *z*, dorsal to ventral with positive values indicating right, anterior, or dorsal and negative values left, posterior, or ventral, respectively. The number of voxels in each cluster reflects contiguous voxels in which *p *<* *.005 after applying appropriate corrections for multiple testing.

a All coordinates reported according to stereotaxic array of Talairach and Tournoux (1988).

**Figure 3:**
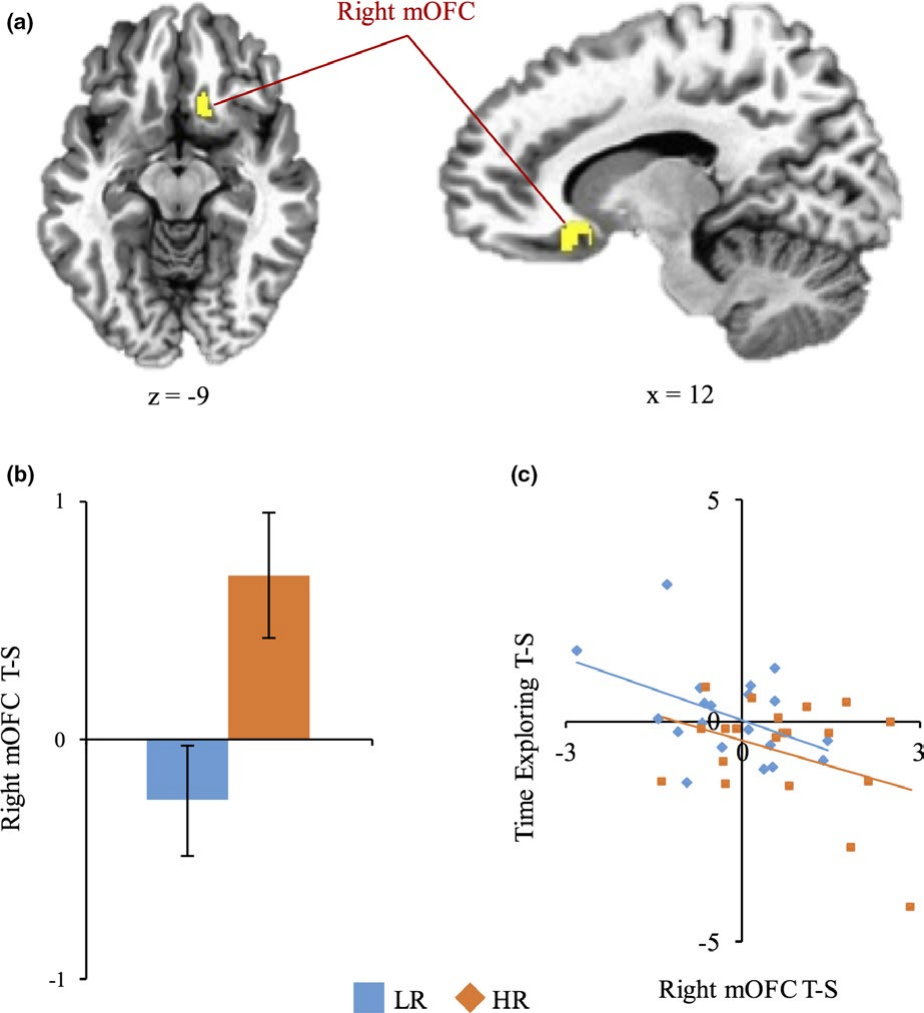
Relative to low‐risk group (LR), high‐risk group (HR) exhibited greater activation in the right medial orbitofrontal cortex (mOFC) during anticipation of unpredictable threat. Greater hemodynamic activity in the mOFC was associated with greater behavioral inhibition (BI). (a) mOFC region of interest mask from which percent signal change (PSC) for threat vs. safe contrast (anticipation of unpredictable threat [AUT]) was extracted. (b) Graph showing PSC for LR and HR groups during AUT [*t*_(38)_ = 2.31, *p* < .01]. (c) Scatterplot indicating a negative relationship between activation in the right mOFC during AUT and time spent exploring threat relative to safe context (BI) in the full sample [*r*_(38)_ = −.49, *p* < .01]. Results shown were corrected for multiple comparisons at *p*_corr_ < 005. Left is left

Whole‐brain voxel‐wise analysis did not reveal any significant differences in BOLD responses between groups for the contrast threat versus safe. Region of interest analyses using extracted PSC from bilateral mOFC revealed significant group differences in the right mOFC, such that the HR group exhibited greater activity as compared to the LR group [*t*_(38)_ = 2.31, *p *<* *.01; Figure 3]. The group differences were not significant for the left mOFC [*t*_(38)_ = .88, *p *=* *.38], or the left or right BNST [*t*_(38)_ = 1.47, *p *=* *.15 and *t*_(38)_ = 0.70, *p *=* *.49, respectively]. We conducted correlational analyses to explore relationships between brain responses and BI and SCRs. Greater right mOFC activity was significantly related to greater BI [Figure 3; *r*_38)_ = −.49, *p < *.01] and a decrease in SCRs [*r*_(38)_ = −.42, *p < *.01]. Left mOFC was unrelated to BI and SCRs in the whole group [*r*_*(*38)_ = −.26, *p = .10* and *r*_*(*38)_ = −.03, *p = *.99, respectively]. BNST activity was unrelated to SCR (Left BNST: *r*_(38)_ = −.16, *p = *.32; Right BNST: *r*_(38)_ = −.20, *p = *.21) and BI (Left BNST: *r*_(38)_ = −.28, *p = *.08; Right BNST: *r*_(38)_ = −.06, *p = *.72).

## 4 DISCUSSION

In this study, we tested the hypothesis that otherwise medically and psychiatrically healthy adults at risk for affective disorders by virtue of family history would exhibit divergent behavioral and neural responses to AUT as compared to individuals with no such family history. The study yielded three major results. First, LR and HR adults exhibited similar levels of subjective anxiety and SCR under conditions of unpredictable threat. Second, as predicted HR adults evidenced greater BI than LR adults under conditions of unpredictable threat. Finally, unpredictable threat resulted in greater activation in the right mOFC in HR adults as compared to LR adults. Together, these results support the view that mOFC plays a role in anticipation of aversive outcomes and regulation of subsequent affective states and behavioral responses, which among healthy HR adults may play a protective role.

The heightened mOFC responses to threat in HR individuals in part replicate findings from other neuroimaging studies with at‐risk children and adolescents, as well as patient populations, which have found dysfunction in prefrontal structures in response to aversive stimuli and negative moods (Chai et al., 2015; Gotlib et al., 2010; Mannie et al., 2008, 2011; McCabe et al., 2012). Evidence in primates and humans indicates that, as a result of its significant reciprocal projections with subcortical structures, the OFC sends information about threat probability and the related costs to the amygdala, BNST, and nucleus accumbens, which in turn modulate fear and anxiety responses, including freezing (Kalin, Shelton, & Davidson, 2007; Kalin, Shelton, Fox, Oakes, & Davidson, 2005; Motzkin et al., 2015). In primates, lesions in the OFC result in significant decreases in threat‐induced freezing (i.e., BI) and a general reduction in anxiety, possibly through modulation of BNST activity (Fox et al., 2010; Kalin et al., 2007). This modulation of BNST activity by OFC has been replicated in a small study of human subjects with focal, bilateral lesions in the vmPFC/OFC, where the lesion patients exhibited significantly lower right BNST activity (Motzkin et al., 2015). Although OFC has weak projections to motor areas, it influences behavior by signaling predictions about threat to regions involved in action selection and execution, including the striatum and cingulate cortex (Grupe & Nitschke, 2013; Rudebeck & Murray, 2014). In this study, mOFC activation related to BI, characterized by less exploratory behavior under conditions of threat relative to safety. This further supports the role of this structure in modulation of behavioral responses to threat.

HR adults in our study did not exhibit elevated subjective anxiety and autonomic responses that have been previously reported among at‐risk children and adolescents in response to threatening stimuli (Craske et al., 2008; Grillon et al., 1998). Furthermore, although AUT resulted in increased activity in bilateral BNST for all participants, we did not observe differences between groups. The increased activation in mOFC, but lack of observed differences in subjective anxiety, SCRs, and BNST suggest that, while HR adults in this study exhibited greater threat expectancy and avoidance behaviors, they appear to successfully regulate their physiological responses to AUT. Therefore, the recruitment of mOFC in this study in HR individuals may serve to promote BI, as well as to decrease subjective and autonomic responses. This is supported by the finding that, across the entire study sample, greater mOFC activation related to lower SCRs during threat versus safe conditions. We propose that heightened OFC activation associated with threat expectancy and cost evaluation, followed by BI may serve as a protective factor of sorts for some individuals. First, this may be an adaptive process that has come about to regulate fear responses. Following increased expectancy of threat, caution and withdrawal could act as strategies to avoid the anticipated threat or minimize its impact, thereby reducing subjective anxiety and autonomic responses. Second, BI may have become reinforced as a result of its calming impact on the affective experiences in the past. By using a behaviorally inhibited response, an individual could draw not only a sense of control over his or her circumstances but also a sense of mastery in dealing with anticipated threats. Third, using the diathesis‐stress model as a reference point, a cautious disposition may serve to protect from excessive risk‐taking and occurrence of negative events in these young adults, thus potentially preventing mood and anxious symptomatology. Finally, avoidance of anticipated negative events is often an adaptive response, particularly in situations where such avoidance does not result in the sacrifice of potential rewards (e.g., avoidance of a poisonous snake would be seen as purely adaptive) (Aupperle & Paulus, 2010). In the current paradigm, there was no incentive for exploratory behavior and, thus, BI could represent an adaptive avoidance response. Therefore, while it may be argued that the patterns observed in this study represent a marker of vulnerability in HR individuals, this may not represent the full picture. We propose that recruitment of mOFC during AUT serves to regulate fear responses by overestimating the likelihood and cost of negative consequences, and propelling the organism to behave cautiously so to prepare for or avoid the threat altogether. Under conditions of excessive and prolonged stress, this mechanism may fail to achieve the desired effect by becoming overly strained and generalized, significantly inhibiting ability to experience desired rewards, in turn leading to withdrawal and diminished positive affect (subsequently leading to diagnosable mood and anxiety disorders). Longitudinal studies examining whether increased mOFC activity during anticipation of threat predicts longer‐term outcomes of HR individuals could help test these hypotheses. If supported, prevention targets for high‐risk individuals may include contextual discrimination (i.e., threat vs. safety), in order to best recognize when BI is adaptive and when it is not.

The following considerations should be kept in mind when interpreting the results of this study. The HR sample in this study consisted of adults between ages 18 and 47, while other studies typically use children, adolescents, and young adults not older than 21. Therefore, differences between our and previous studies may be age related. Additional studies with healthy high‐risk adults are needed to examine how potential risk factors play out later in life. Moreover, while cross‐sectional studies with high‐risk populations like ours can be extremely valuable in bringing to light mechanisms of threat processing and risk for psychopathology, longitudinal designs may be better at delineating whether and how these factors constitute psychopathology, vulnerability, or resilience. In this study, exploration was not associated with increased risk of electric stimulus, and subjects were not given an option of entering or leaving the threatening and safe contexts, thus prohibiting assessment of active avoidant behaviors. Finally, our study did not employ groups with mood or anxiety disorders, prohibiting us to conclude whether our results more closely resemble healthy adults without risk or adults with psychopathology.

## 5 CONCLUSIONS

Results from this study indicate that medically and psychiatrically healthy adults with family history of affective disorders showed increased activation in the right mOFC, as well as greater BI, during anticipation of aversive stimuli. Furthermore, we observed a positive relationship between mOFC activity and BI, both of which were inversely related to autonomic responses. The results therefore support the view that mOFC is part of a neural circuit involved in processing anticipation of aversive outcomes and regulation of subsequent affective states and behavioral responses. Although group differences in this study may point to a marker of vulnerability for affective disorders, absence of symptoms in this sample, as well as undifferentiated subjective and autonomic responses potentially suggest a protective, resilient mechanism.

## CONFLICT OF INTEREST

The authors report no conflict of interest.
