# Supplementary material for: Recruitment of orbitofrontal cortex during unpredictable threat among adults at risk for affective disorders
Source: Brain Behav. 2017 Jul 11;7(8):e00757. doi: 10.1002/brb3.757 (PMC5561318; doi:10.1002/brb3.757)
Supplement: Supplementary file 1 [file BRB3-7-e00757-s001.docx]

**APPENDIX**

**Appendix A. Supplementary Methods**

*Participants*

One hundred and twenty-eight individuals were recruited for the present study, including 78 psychiatrically and medically healthy individuals. Of these 78, 24 (30.1%) were classified as high risk individuals based on family history of psychopathology, of which 20 were included in the present study after undergoing data quality control. High risk participants had first-degree relatives (e.g., mother; n=17) and/or second-degree relative (e.g., grandfather; n=3) with a mood disorder, consisting of major depressive disorder in all but three cases where the relative had bipolar disorder (one first-degree, two second-degree relatives). In four cases, relatives with MDD also had a comorbid anxiety disorders. Exclusion of high risk participants with relatives with bipolar disorder did not change the results; therefore, they were kept in all analyses.

*General Procedure*

Prior to entering the scanner, participants received task instructions, completed questionnaires, and underwent sensor application to allow for nociceptive flexion reflex/pain threshold testing and online measurement of skin conductance responses (SCRs). Previous research has shown that subjective pain is highly correlated with an index of spinal nociception, the nociceptive flexion reflex (NFR), a lower limb withdrawal response evoked by Aδ fibers (Chan & Dallaire, 1989). Therefore, pain threshold testing procedures were carried out prior to scanning to achieve a level of stimulation necessary to reliably elicit the NFR, as well as a subject’s subjective rating of the stimulus as painful, a rating of 50 on the 0-100 Stimulus Intensity scale. Following threshold testing, participants practiced navigating a non-task virtual context for two minutes in a full-scale MRI simulator (mock scanner) under no threat of receiving stimulation. To minimize unwanted movement during scanning and in response to electric stimulations, participants wore velcro straps over the hips and left ankle. Additionally, to minimize head motion, stabilizing head foam pads were placed around each participant’s head. Participants first completed anatomical and resting-state scans, followed by a 4-min practice scan during which they explored the task contexts according to task instructions. A single electric stimulation in the threat context was delivered during the practice scan. Immediately following the practice scan, participants completed a contingency awareness test designed to test the accurate understanding of experimental contingencies.

*Contextual Stimuli*

The visual stimuli, including the two computer-simulated rooms (contexts), were generated by a customized software application (Vizard Virtual Reality Software Toolkit, WorldViz; Santa Barbara, CA; see Figure 1) installed on a Hewlett-Packard desktop running Windows XP (Hewlett-Packard, L.P., Palo Alto, CA). The two virtual contexts, visually distinguishable by virtue of their distinctive purple- or peach-colored walls and furniture, were identical in layout and size. During each context presentation participants navigated the entirety for each room using a four-button response pad that allowed for forward, backward, left, and right movements.

*Anticipation of Unpredictable Threat (AUT) Task*

Prior to scanning, participants were informed that, during the task, they were to virtually explore two computer-simulated rooms projected onto a screen in the scanner, and that later they would be asked what they learned about each room. Half the participants were told that, whenever they were in the “purple room,” they could receive a stimulation on the ankle at any time, and that, whenever they were in the “peach room,” they would never receive a stimulation; the other half were given the opposite instructions. During each of 4 fMRI scans, 5 threat and 5 safe contexts were each semi-randomly presented for a duration of 18 seconds followed by an interstimulus interval (ISI) of 14-18 seconds. Order of scan presentation (e.g., 1, 2, 3, 4; 2, 3, 4, 1…) was counterbalanced across participants. An unpredictable (i.e., unsignaled) electrical stimulation served as an unconditioned stimulus (US) and was delivered during 1-2 threat contexts each fMRI scan for a total of 5 unconditioned stimuli (range 3-16 sec post-context onset; mean onset=9.6 sec). No US was administered during the safe context. During the ISI, participants performed a low-level vigilance task in which they fixated on a central plus sign and pressed a button on the response pad anytime it changed color (1-2 times per ISI). Following each threat context in which an electric stimulus was administered, participants rated the intensity of the stimulus received during the ISI.

At the conclusion of each 350-second fMRI scan, participants also retrospectively rated how fearful they were in the threat and safe contexts (Figure 1). Although the AUT task shares similarities with unsignaled contextual fear conditioning where subjects gradually learn context-US associations through direct experience (Alvarez, Biggs, Chen, Pine, & Grillon, 2008), the AUT task involves explicit verbal instruction about threat-safe contingencies from the outset. Therefore, it was expected that throughout the task participants would engage in sustained anticipation of unpredictable threat during the threat context compared to the safe context.

*Electric Stimuli and Skin Conductance Acquisition and Assessment*

Electric stimulation during threshold testing and scanning were delivered using two MRI-compatible Ag-AgCl stimulating surface electrodes (2 cm inter-electrode distance) attached to the left ankle over the retromalleolar pathway of the sural nerve, 2 cm posterior to the malleolus (Roy, Piche, Chen, Peretz, & Rainville, 2009). A Digitimer DS7A stimulator (Hertfordshire, England) generated electric stimuli that were triggered by the presentation computer and the Agilent 33220A waveform generator (Santa Clara, CA). Two MRI-compatible Ag-AgCl surface electrodes (3 cm inter-electrode distance) measuring electromyographic (EMG) activity over the left biceps femoris muscle, 10 cm superior to the popliteal fossa, recorded the NFR. Skin at the location of stimulating and recording electrodes was cleaned with NuPrep to reduce impedance to less than 5kΩ and 10kΩ, respectively. To minimize habituation and predictability during NFR testing, electrical stimulation of the sural nerve was repeated according to a variable 8-12 seconds interstimulus interval. Each stimulation consisted of a train of 5, 1-ms rectangular pulses with a 3-ms interpulse interval. Using an ascending staircase method, stimulation intensity started at 1mA and increased in 2mA increments until stimulation subjectively deemed painful was achieved and a reliable NFR is detected. To eliminate potential interference from the RII reflex, startle reactions, and voluntary movement, a detected NFR was defined as a mean EMG response in the 90- to 150-ms poststimulation interval that exceeds the mean EMG activity during the 60-ms prestimulation baseline interval by at least 1.5 standard deviations (SD) (Dowman, 1992). Use of the 1.5 SD cut-point allows for 88% specificity and 82% sensitivity to detect an NFR (Rhudy & France, 2007). The final stimulation used during the AUT task was set at 1.2 times the level at which both the pain threshold and NFR were obtained, with maximum stimulation intensity never exceeding 40mA for the safety of the participant.

Skin conductance responses (SCR) were recorded during each fMRI scan with MRI-compatible Ag-AgCl electrodes placed on the medial side of the right foot over the abductor hallucis muscle (Fowles et al., 1981), and using a Biopac Systems electrodermal activity module. Offline data analysis of SCRs was performed using the general linear convolution model-based analysis of waveforms (Bach, Daunizeau, Friston, & Dolan, 2010; Bach, Flandin, Friston, & Dolan, 2009) as implemented in SCRalyze software (2.1.6b, scralyze.sourceforge.net) to estimate the mean response amplitude for threat and safe conditions, respectively. This approach has been found to be more sensitive than other traditional response estimates, where evoked responses relative to a pre-event baseline are measured (Bach et al., 2009). As per Bach and colleagues’ (2009) recommendation, the SCR data were normalized to avoid potential bias due to inter-subject differences in response amplitude originating from differences in skin properties or other peripheral factors. However, we used non-normalized data for group comparisons to avoid any variance being removed between groups secondary to z-transformation. The SCR analysis included only threat trials in which no US was delivered, a comparable number of safe trials, and modeling of all extraneous events including US delivery. Thus, the analysis of SCRs to threat contexts were unbiased by intermittent US delivery.

*Physiological Monitoring*

Physiological data, including the electromyogram, heart and respiration rates, exhaled carbon dioxide (CO2) concentration, and electrodermal activity were recorded throughout scanning. A belt placed over the subject’s torso measured respiratory changes, while a finger pulse oximeter was used to record heart rate. Exhaled end-tidal CO2 concentration was recorded using nasal cannulae and plastic tubing connected to a standard transducer. Amplification, filtering, and recording of physiological data was done using the BIOPAC Systems, Inc. acquisition system and software (AcqKnowledge, 4.1), received via the MRI-compatible and radiotranslucent (metal-free and not absorbing radio frequency (RF) power) electrodes and leads, and isolated RF filtered cables.

*Data Preprocessing*

Functional image preprocessing and analysis was performed using AFNI (<http://afni.nimh.nih.gov/afni>). In addition, Advanced Normalization Tools (ANTS; see <http://stnava.github.io/ANTs/>) was used to optimize spatial alignment of functional data to the TT_N27 T1-weighted template. Because ANTs uses symmetric diffeomorphic image registration to perform nonlinear warping on a voxel-by-voxel basis rather than warping all voxels by the same parameters, as is typically done with linear transformation, nonlinear spatial normalization with ANTs can produce more accurate alignment especially at smaller brain structures, and has been found to outperform other nonlinear methods (Avants, Epstein, Grossman, & Gee, 2008; Klein et al., 2009). After discarding the first 5 volumes of each EPI time course to allow the fMRI signal to achieve steady state, slice timing correction was performed using the initial slice acquired at the beginning of each volume as a reference. The anatomical image was aligned to the first EPI image with align_epi_anat.py script in AFNI. The aligned anatomical image, which was resampled to the same resolution as the EPI image, was warped to the TT_N27 T1-weighted template using ANTs. The template image was resampled to 1.875 mm^3^ voxel size beforehand, which determined the size of the normalized EPI image. After realigning the EPI images to the first volume for motion correction, the EPI images were normalized to the template image using the warping parameters obtained from the anatomy to template alignment. To reduce noise in the images without losing substantial spatial resolution, the EPI data were smoothed with a small 1.875 mm FWHM Gaussian kernel. The signal intensity of each EPI volume was then normalized so as to reflect percent signal change from the mean intensity of each voxel across the time course.

Functional imaging data at the single subject level were analyzed with 3dREMLfit, a regression program that estimates the serial correlation structure of the noise with an ARMA(1, 1) model, and uses the subsequent temporal correlation matrix to estimate beta parameters using a generalized least squares (GLSQ) method. The GLSQ approach typically produces beta values with smaller variance. The regression model included regressors for each task context as well as regressors of non-interest to account for head motion, signal trends, electric stimulations, stimulation intensity ratings, fixation color changes, end-tidal CO2, and navigation behavior. To give the shape of the blood oxygenation level-dependent (BOLD) response maximum flexibility, each task context was modeled as the sum of piecewise linear B-spline basis functions or tent functions. Fifteen tent functions covering 30s were used to account for the full extent of each context (0-18 s) and the recovery of the BOLD response following context offset. For the contrast of threat context versus safe context, the voxelwise analysis included only regressors for the ten time points (0 - 18s) spanning each context. The first time point (0 s) was assumed to have zero magnitude to account for the expected delay in the BOLD response to context onset. The time points following context offset were treated as regressors of non-interest. The analysis was designed in such a way that the results were not biased by the delivery of US. Specifically, responses to threat context included only the trials in which shock was not delivered. Only safe context trials closest in proximity to the unreinforced threat trials were used in order to allow for an equal number of trials in the threat versus safe contrast.

**Supplementary References**

Alvarez, R. P., Biggs, A., Chen, G., Pine, D. S., & Grillon, C. (2008). Contextual fear conditioning in humans: cortical-hippocampal and amygdala contributions. *J Neurosci, 28*(24), 6211-6219. doi:10.1523/JNEUROSCI.1246-08.2008

Avants, B. B., Epstein, C. L., Grossman, M., & Gee, J. C. (2008). Symmetric diffeomorphic image registration with cross-correlation: evaluating automated labeling of elderly and neurodegenerative brain. *Medical Image Analysis, 12*(1), 26-41.

Bach, D. R., Daunizeau, J., Friston, K. J., & Dolan, R. J. (2010). Dynamic causal modelling of anticipatory skin conductance responses. *Biological psychology, 85*(1), 163-170. doi:10.1016/j.biopsycho.2010.06.007

Bach, D. R., Flandin, G., Friston, K. J., & Dolan, R. J. (2009). Time-series analysis for rapid event-related skin conductance responses. *J Neurosci Methods, 184*(2), 224-234. doi:10.1016/j.jneumeth.2009.08.005

Chan, C. W., & Dallaire, M. (1989). Subjective pain sensation is linearly correlated with the flexion reflex in man. *Brain Research, 479*(1), 145-150. doi:10.1016/0006-8993(89)91344-9

Dowman, R. (1992). Possible startle response contamination of the spinal nociceptive withdrawal reflex. *Pain, 49*(2), 187-197.

Fowles, D. C., Christie, M. J., Edelberg, R., Grings, W. W., Lykken, D. T., & Venables, P. H. (1981). Publication recommendations for electrodermal measurements. *Psychophysiology, 18*(3), 232-239.

Klein, A., Andersson, J., Ardekani, B. A., Ashburner, J., Avants, B., Chiang, M.-C., . . . Parsey, R. V. (2009). Evaluation of 14 nonlinear deformation algorithms applied to human brain MRI registration. *Neuroimage, 46*(3), 786-802. doi:10.1016/j.neuroimage.2008.12.037

Rhudy, J. L., & France, C. R. (2007). Defining the nociceptive flexion reflex (NFR) threshold in human participants: A comparison of different scoring criteria. *Pain, 128*(3), 244-253. doi:10.1016/j.pain.2006.09.024

Roy, M., Piche, M., Chen, J.-I., Peretz, I., & Rainville, P. (2009). Cerebral and spinal modulation of pain by emotions. *PNAS Proceedings of the National Academy of Sciences of the United States of America, 106*(49), 20900-20905. doi:10.1073/pnas.0904706106
