# Supplementary figures and images for: Recruitment of orbitofrontal cortex during unpredictable threat among adults at risk for affective disorders
Source: Brain Behav. 2017 Jul 11;7(8):e00757. doi: 10.1002/brb3.757 (PMC5561318; doi:10.1002/brb3.757)

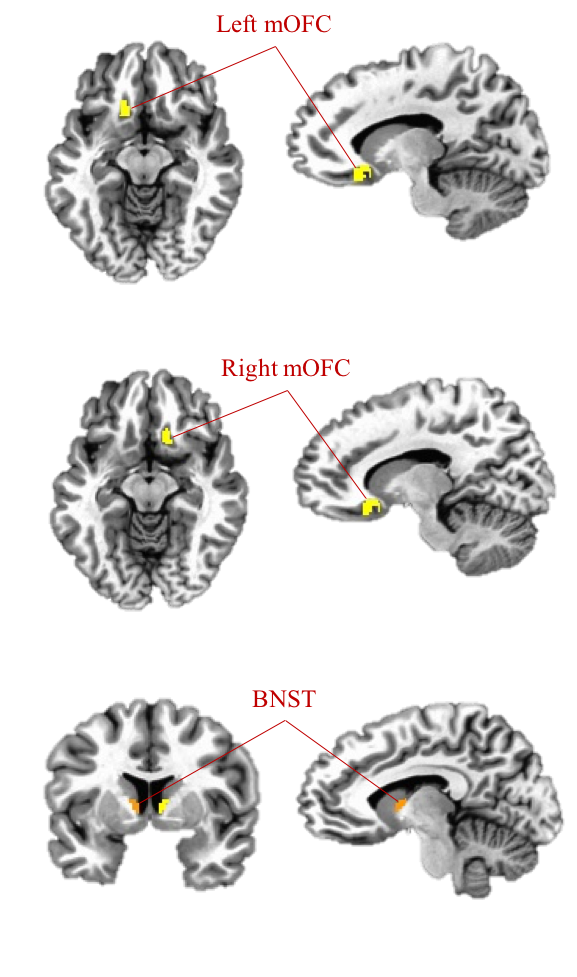

Supplement: Supplementary file 2 [file BRB3-7-e00757-s002.png]
